# Supplementary material for: Neuromodulatory subcortical nucleus integrity is associated with white matter microstructure, tauopathy and APOE status
Source: Nat Commun. 2024 Jun 3;15:4706. doi: 10.1038/s41467-024-48490-z (PMC11148077; doi:10.1038/s41467-024-48490-z)
Supplement: Supplementary file 1 — Supplementary Information [file 41467_2024_48490_MOESM1_ESM.pdf]

Supplementary Materials

Neuromodulatory subcortical nucleus integrity is associated with white matter microstructure, tauopathy and APOE status

Alfie Wearn, Stéfanie A. Tremblay, Christine L. Tardif, Ilana R. Leppert, Claudine J. Gauthier, Giulia Baracchini, Colleen Hughes, Patrick Hewan, Jennifer Tremblay-Mercier, Pedro Rosa-Neto, Judes Poirier, Sylvia Villeneuve, Taylor W. Schmitz, Gary R. Turner & R. Nathan Spreng

for the PREVENT-AD Research Group

Isodendritic core masks

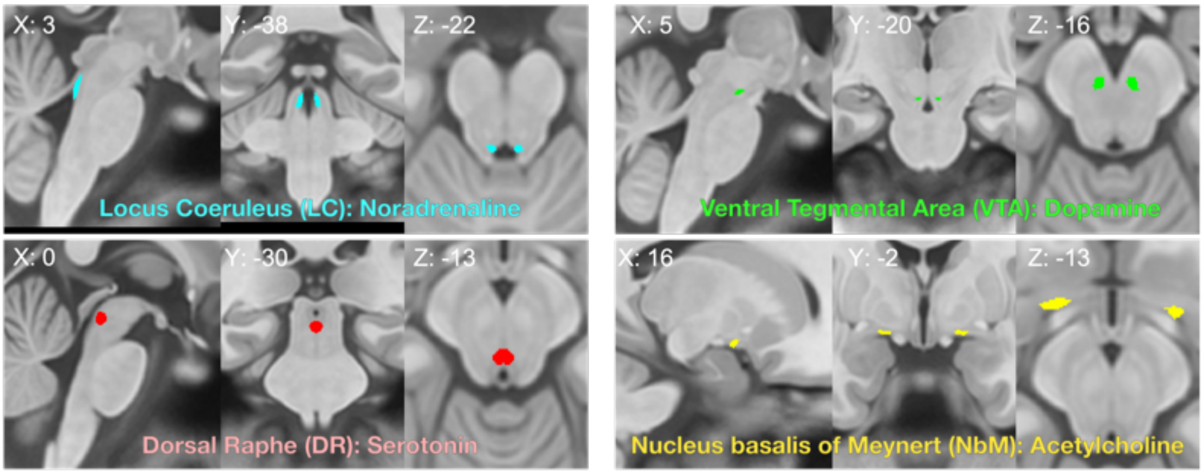

Supplementary Fig. 1 | ***Isodendritic core nuclei region-of-interest masks****. Locus Coeruleus, Dorsal Raphe, Ventral Tegmental Area, and Nucleus Basalis of Meynert shown in MNI space (ICBM 2009b 0.5mm asymmetric) with MNI coordinates. Each nucleus is also labelled with its primary associated neuromodulator.*

Discrepancies in Dorsal Raphe localization

While validated atlases were used to define locus coeruleus, ventral tegmental area and nucleus basalis of Meynert, we elected in this study to use a manually defined mask of the dorsal raphe. Although publicly available masks of dorsal raphe do exist [6–8], we observed large discrepancies between their spatial profiles, with little overlap between them in some cases. For example, the mask of Bianciardi et al.[7] (thresholded at 50%) extends considerably more rostrally, up to the level of the cerebral aqueduct and superior colliculus, than those of Levinson et al.[6] (thresholded at 50%) and Edlow et al.[8] (Supplementary Fig. 2). Other studies examining the dorsal raphe define a ~150mm^3 region centered on MNI coordinates x: 0, y: -27 / -31, z: -9 [3,9,10]. This ROI, defined in relation to histological sources, is then refined in relation to serotonin transporter (5-TT) PET images, which highlight serotonergic neurons in the dorsal and median raphe nuclei. This places the location of dorsal raphe ROI fairly rostral, in line with the cerebral aqueduct. This is similar to the mask of Bianciardi et al.[7], but does not overlap with the masks of Levinson et al.[6] and Edlow et al.[8].

These discrepancies may arise as a result of methodology. Studies which place DR more rostrally tend to rely on direct histological reference and/or co-localization with 5-TT PET and may therefore be more localized to the serotonergic neurons in the rostral supratrochlear nucleus of the dorsal raphe, assuming caution is take to avoid accidentally including the serotonin-rich median raphe nucleus[11]). This supratrochlear nucleus, critically, is where neurofibrillary tangles have been identified [12], and is the particular subregion of interest for the present study. Studies which localize dorsal raphe more caudally [6,8] tend to rely on diffusion imaging and may be more sensitive to identifying cells with more densely packed afferent and

efferent fibres, regardless of the subnucleus. Diffusion-based methods may be more sensitive to localizing more caudal sections of DR, at the expense of the more rostral subnuclei.

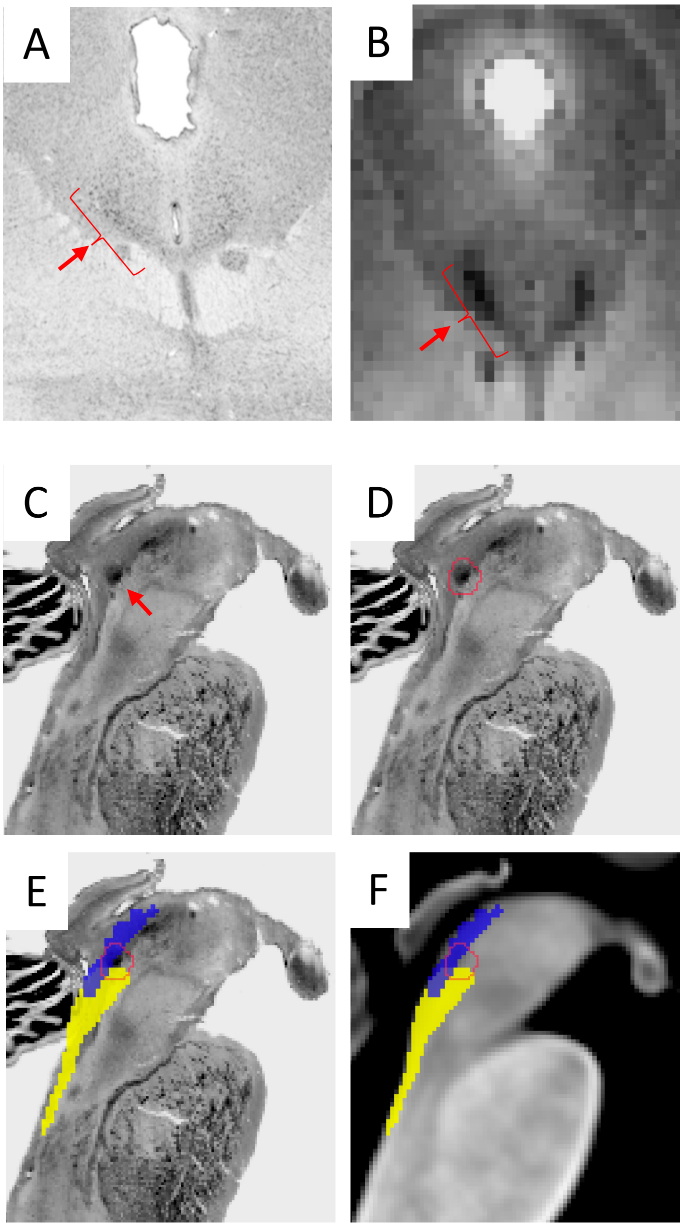

Supplementary Fig. 2 | Location of dorsal raphe ROI on in MNI space. A: Coronal slice of BigBrain (y=-8.25mm). The 'wings' of the supratrochlear nucleus of dorsal raphe are clearly visible, highlights my red arrow for right hemisphere. B: Coronal slice of MNI-space BigBrain (MNI-space y=-30.5). The view is analogous to that in panel A, with the same supratrochlear dorsal raphe 'wing' highlighted. C: Sagittal view of MNI-space BigBrain (x=1), with arrow highlighting the right-hemisphere dorsal raphe 'wing', visible as a dark spot in this view, in line with the inferior colliculus. D: The same view as in C, but with a red circle highlighting our manually-defined dorsal raphe ROI. E: The same view as in D, but with the extra overlays of Biancardi 'Brainstem Navigator' dorsal raphe ROI (blue, thresholded at 50%) and the Levinson et al. dorsal raphe ROI (yellow, thresholded at 50%). F: The same sagittal view as in E, but the image shows T1-weighted MNI-template instead of BigBrain.

Registration Mask

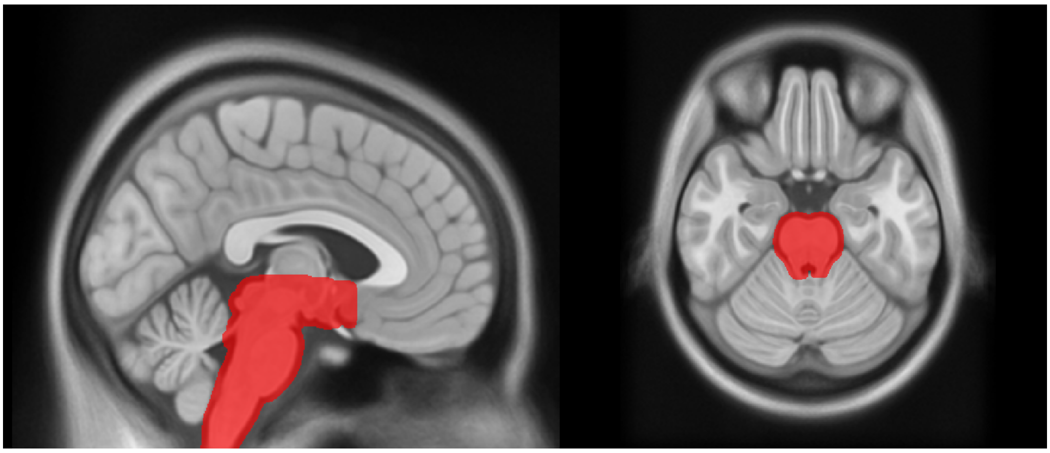

Supplementary Fig. 3 | Mask used to aid registration of subjects' anatomical scans into MNI space.

Pontine region (control)

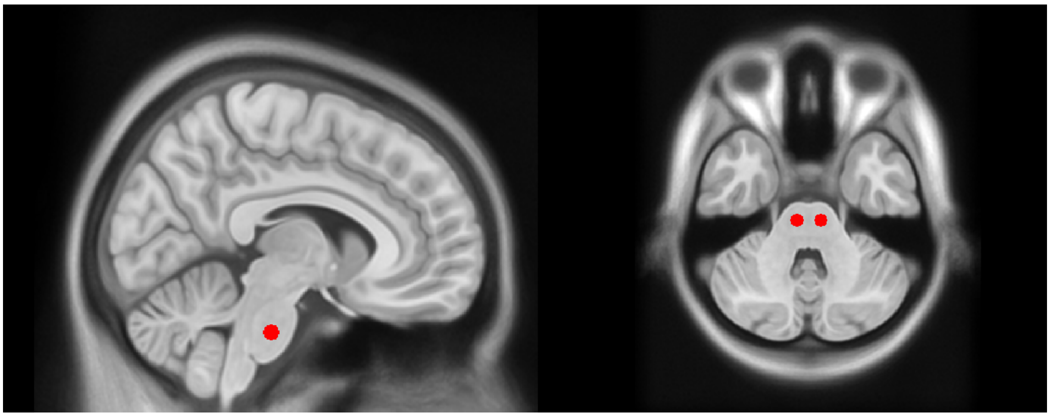

Supplementary Fig. 4 | Bilateral pontine ROI used as a control region in PLS analysis, shown in red, overlayed on MNI 2009b brain.

White Matter mask

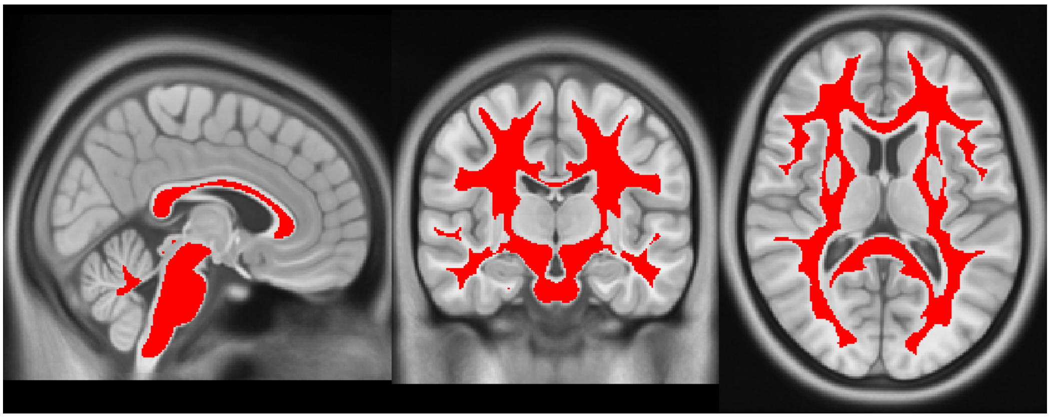

Supplemental Fig. 5 | White matter mask. This mask was used to restrict the voxel-wise PLS correlation only to white matter voxels within the NODDI maps. The mask was thresholded at 0.95 after averaging all individual probabilistic masks from all subjects. Red voxels show the presence of the mask overlaid over a standard-space T1-weighted image.

Supplementary Table 1 | Demographics of groups with and without available CSF data.

	**With CSF**	**No CSF**
n	93	40
Age (years)	67.5 ± 5.58	68.8 ± 4.58
Sex (female)	66 (69%)	30 (75%)
Education (years)	15.2 ± 0.26	15.8 ± 0.34

Supplementary Table 2 | Average MPM values for each ROI, split by APOE4 carrier status.

**MPM**	**Group**	LC	DR	VTA	NbM	Pontine region
R1 (1/s)	**All subjects**	**0.61 ± 0.03**	**0.58 ± 0.03**	**0.64 ± 0.04**	**0.65 ± 0.03**	**0.79 ± 0.04**
	APOE4+	0.61 ± 0.03	0.58 ± 0.03	0.65 ± 0.03	0.65 ± 0.03	0.79 ± 0.04
	APOE4-	0.61 ± 0.03	0.58 ± 0.03	0.64 ± 0.04	0.65 ± 0.03	0.79 ± 0.04
MTsat (p.u.)	**All subjects**	**0.90 ± 0.10**	**0.84 ± 0.09**	**0.93 ± 0.11**	**0.91 ± 0.07**	**1.44 ± 0.12**
	APOE4+	0.92 ± 0.10	0.85 ± 0.08	0.96 ± 0.12	0.92 ± 0.08	1.43 ± 0.12
	APOE4-	0.89 ± 0.10	0.84 ± 0.10	0.92 ± 0.09	0.91 ± 0.07	1.45 ± 0.12
R2* (1/s)	**All subjects**	**15.0 ± 2.07**	**14.8 ± 1.75**	**19.0 ± 2.54**	**24.9 ± 2.48**	**20.2 ± 2.48**
	APOE4+	15.1 ± 1.88	15.0 ± 1.66	19.0 ± 2.51	24.9 ± 2.41	19.9 ± 2.81
	APOE4-	14.9 ± 2.19	14.7 ± 1.82	19.1 ± 2.56	24.9 ± 2.55	20.4 ± 2.28
PD (p.u.)	**All subjects**	**76.1 ± 1.42**	**76.1 ± 1.38**	**76.6 ± 1.84**	**77.5 ± 1.28**	**68.8 ± 1.44**
	APOE4+	76.0 ± 1.40	76.1 ± 1.30	76.1 ± 1.71	77.4 ± 1.05	68.9 ± 1.09
	APOE4-	76.1 ± 1.44	76.1 ± 1.42	76.6 ± 1.93	77.6 ± 1.41	68.8 ± 1.19

PLS Covariance patterns between APOE4 groups

All associations between isodendritic core measures of microstructure and white matter NODDI parameters are described below. All data are shown in Figure 6 of the main manuscript. For clarity, each MPM measure is written as a subscript note as it relates to each Isodendritic core region. For example, proton density in the locus coeruleus is written as $LC_{PD.}$

Pattern 1

This pattern highlighted a widespread pattern of voxels across the entire brain. The associations across these voxels for each APOE4 subgroup were as follows:

APOE4-

LC_{PD} was negatively associated with FW.

VTA_{PD} was strongly positively associated with NDI. VTA_{R1} and VTA_{R2*} were positively associated with ODI.

NbM_{R1} was positively associated with ODI.

APOE4+

LC_{R1} and LC_{MTsat} were positively associated with NDI, ODI and FW across this broad area of white matter. LC_{R2*} was also positively associated with NDI and ODI.

DR_{R1} was positively associated with NDI, ODI and FW. DR_{MTsat} was positively associated with ODI and FW. DR_{R2*} was positively associated with NDI and ODI. DR_{PD} was negatively associated with ODI and FW.

VTA_{R1} was positively associated with FW. VTA_{R2*} was both strongly positively associated with NDI.

NbM_{R1} was positively associated with NDI and FW. NbM_{MTsat} was positively associated with both NDI and ODI.

Pattern 2

This pattern dissociated limbic tracts such as cingulum bundle from brainstem efferent tracts including corona radiata. The associations across the brainstem efferent regions for each APOE4 subgroup are described below. The described associations were all inverted in the limbic regions:

APOE4-

LC_{R1} was positively associated with ODI in the brainstem-efferent regions. LC_{R2*} was positively with NDI.

DR_{R1} and DR_{MTsat} were positively associated with ODI in the brainstem-efferent regions.

VTA_{R1} and VTA_{R2*} were positively associated with both ODI and FW in brainstem-efferent regions.

NbM_{R1} was negatively associated with NDI and positively associated with ODI in the brainstem-efferent regions.

The described associations were all inverted in the limbic areas of the white matter.

APOE4+

LC_{R1} and LC_{MTsat} were both negatively associated with NDI and positively associated with ODI and FW in the brainstem-efferent regions. LC_{R2*} was negatively associated with NDI and positively associated with ODI in these regions.

DR_{R1}, DR_{MTsat} and DR_{R2*} were all negatively associated with NDI and FW and positively associated with ODI in the brainstem-efferent regions. DR_{PD} was negatively associated with ODI in these regions.

VTA_{MTsat} was positively associated with ODI and negatively associated with FW in the brainstem efferent regions. VTA_{R2*} was negatively associated with NDI and positively associated with ODI in this region. VTA_{PD} was negatively associated with both NDI and FW.

NbM_{R1} and NbM_{MTsat} were both negatively associated with NDI and positively associated with ODI in the brainstem-efferent regions.

Pattern 3

This pattern dissociated the cingulum from peripheral posterior white matter and brainstem efferent tracts. The associations across the cingulum regions for each APOE4 subgroup are described below. The described associations were all inverted in diffuse areas in the peripheral posterior white matter and brainstem efferent tracts:

APOE4-

LC_{R1} and LC_{MTsat} were negatively associated with NDI, ODI and FW in the cingulum. LC_{R2*} was negatively associated only with FW in the cingulum. LC_{PD} was positively associated with NDI, ODI and FW in the cingulum.

DR_{R1}, DR_{MTsat} and DR_{R2*} were negatively associated with NDI, ODI and FW in the cingulum. DR_{PD} was positively associated with NDI, ODI and FW in the cingulum.

VTA_{MTsat} was negatively associated with ODI in the cingulum.

NbM_{MTsat} was negatively associated with NDI and FW in the cingulum. NbM_{PD} was negatively associated with ODI in the cingulum.

APOE4+

VTA_{R1} and VTA_{R2*} were negatively associated with FW in the cingulum. VTA_{MTsat} and VTA_{PD} were positively associated with FW in cingulum. VTA_{MTsat} was also positively associated with NDI in this region.

NbM_{R1} was positively associated with ODI in the cingulum. NbM_{PD} was negatively associated with FW in the cingulum.

Pattern 4

This pattern dissociated the corpus callosum and hippocampal cingulum from superior longitudinal fasciculus. The associations across the corpus callosum and hippocampal cingulum for each APOE4 subgroup are described below. The described associations were all inverted in diffuse areas in the superior longitudinal fasciculus:

APOE4-

LC_{R1} and LC_{MTsat} were negatively associated with ODI in the corpus callosum and hippocampal cingulum. LC_{R2*} was positively associated with FW in these tracts. LC_{PD} was negatively associated with ODI in these tracts.

DR_{R1}, DR_{MTsat} and DR_{R2*} were negatively associated with ODI in the corpus callosum and hippocampal cingulum. DR_{PD} was positively associated with ODI in these tracts.

VTA_{R1} and VTA_{R2*} were negatively associated with ODI and FW in the corpus callosum and hippocampal cingulum. VTA_{MTsat} was positively associated with FW in these tracts.

NbM_{R1} was negatively associated with ODI and FW in the corpus callosum and hippocampal cingulum. NbM_{MTsat} was negatively associated with ODI in these tracts. NbM_{R2*} was negatively associated with FW and NbM_{PD} was positively associated with FW in these tracts.

APOE4+

LC_{R1} and LC_{MTsat} were positively associated with ODI in the corpus callosum and hippocampal cingulum. LC_{MTsat} was also positively associated with FW in these tracts. LC_{PD} was negatively associated with NDI, ODI and FW in these tracts.

DR_{MTsat} was positively associated with NDI in the corpus callosum and hippocampal cingulum. DR_{MTsat} and DR_{R2*} were positively associated with ODI in these tracts.

VTA_{PD} was positively associated with ODI in the corpus callosum and hippocampal cingulum.

NbM_{R1} and NbM_{R2*} were negatively associated with FW in the corpus callosum and hippocampal cingulum.

Correlations between brain scores and CSF pTau in APOE4 groupwise PLS

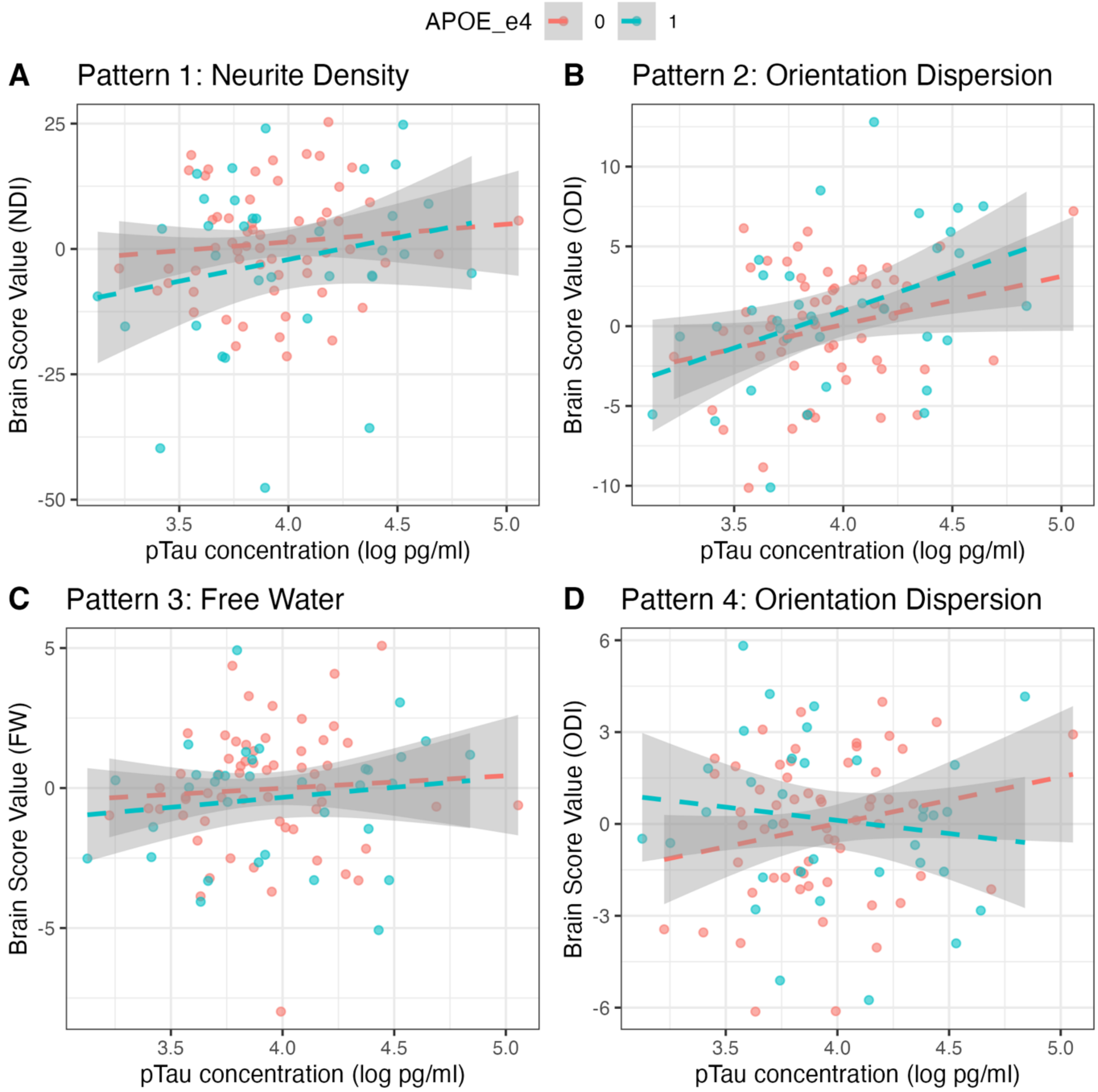

*Supplementary Fig. 6 | Correlations between brain scores and CSF pTau in APOE4 groupwise analysis. Regression statistics are as follows. A) main effect of tau: β=-0.073 p=0.611, tau*APOE4 interaction: β=-0.161 p=0.448, B) main effect of tau: β=0.270 p=0.060, tau*APOE4 interaction: β=0.144 p=0.478, C) main effect of tau: β=-0.058 p=0.697, tau*APOE4 interaction: β=-0.086 p=0.686, D) main effect of tau: β=0.205 p=0.145, tau*APOE4 interaction: β=-0.309 p=0.122. For all tests, n=93 (59 APOE4-, 34 APOE4+). Source data are provided as a Source Data file.*

Testing voxelwise relationship between CSF pTau and NODDI measures

We found that participant brainscores for LV2 of the primary analysis (Fig. 3), which quantitatively express the relationship between IdC and white matter microstructure, was related to CSF pTau. This was further validated in a supplemental analysis where we directly assessed the association between white matter microstructure and CSF pTau.

PLS analysis revealed a significant correlation between log-pTau and age-adjusted NODDI indices, revealing a single significant LV: 43% crossblock covariance explained, p=0.031 (Supplemental Fig. 7). The pattern included regions of corona radiata where pTau is positively related to ODI and negatively related to NDI and FW (shown in yellow). The pattern also includes regions of cingulum bundle and longitudinal fasciculus as well as temporal lobe white matter where pTau is negatively related to ODI and positively related to NDI and FW (shown in blue).

These supplementary results reveal that the spatial topography relating IdC microstructure with white matter microstructure (main analysis LV2), corresponds with the spatial topography relating CSF pTau to white matter microstructure (Supplemental Fig. 7C). This correspondence supports the interpretation that LV2 is a pathological pattern related to AD.

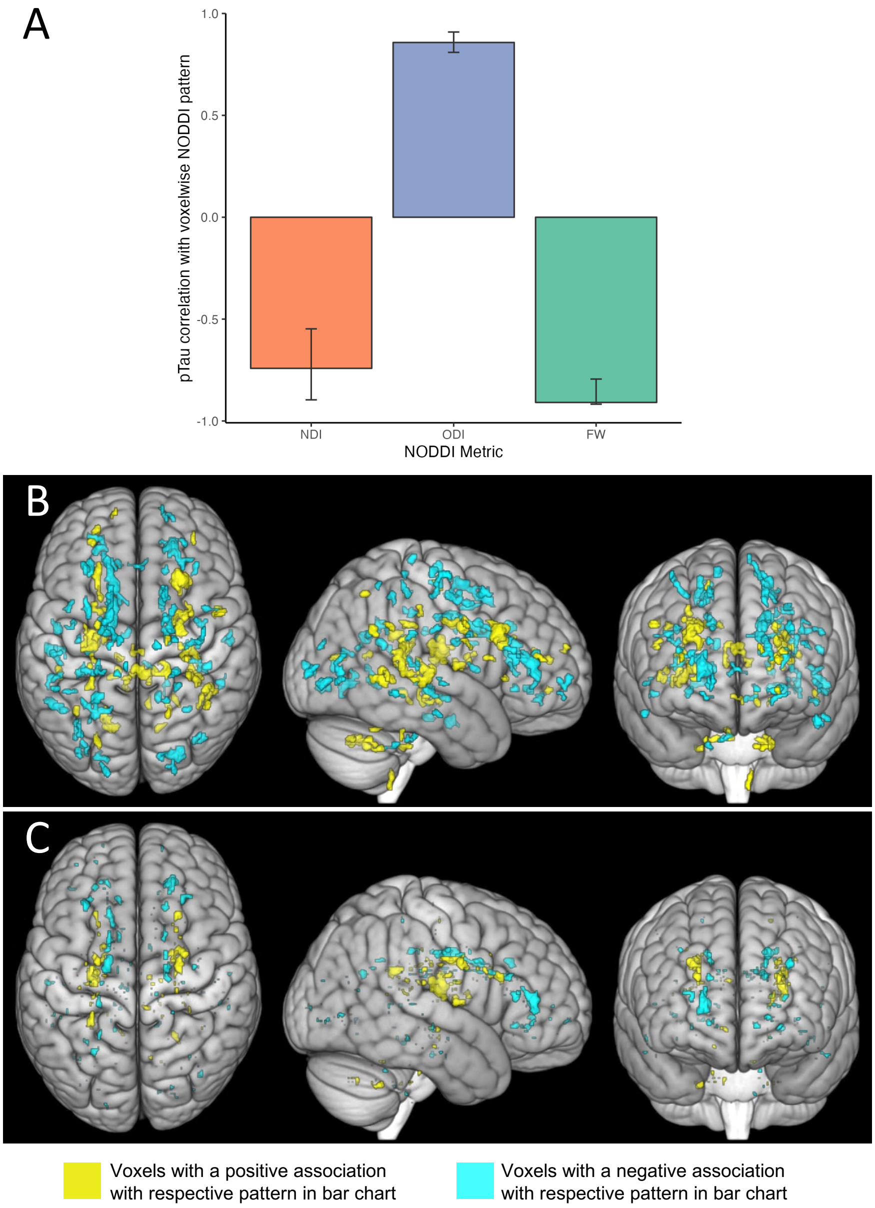

Supplemental Fig. 7 | PLS correlation results between log-pTau CSF concentration and NODDI measures in white matter. A) The strength and direction of the relationship between pTau and each respective NODDI map. Error bars show 95% confidence intervals from 1000 bootstrapped samples. The midpoint of each bar represents the correlation using the full dataset. B) The yellow voxels indicate a positive relationship with the pattern shown in (B). The blue voxels indicate a negative relationship. Only voxels with a bootstrap ratio of >|2| are colored. C) The spatial overlap of patterns shown in panel B and LV2 of the primary analysis (figure 4 of main manuscript). NDI = Neurite Density Index, ODI = Orientation Dispersion Index, FW = Free water fraction. Source data are provided as a Source Data file.

Interpretation of DR microstructure relation to pTau

In most effects shown, we saw patterns that indicate lower R1 is an indicator of poor integrity (e.g. low R1 is associated with older age, as well as lower NDI across the white matter as seen in Pattern 1). However, in Figure 2 we show a positive correlation of R1 in DR with CSF ptau concentration. One explanation for this could be that R1 is directly picking up deposition of neurofibrillary tangles, given their macromolecular structure. Further experimentation would be required to probe this effect further, but we highlight it as an area of future interest to fully dissociate effects of age from Alzheimer's disease pathology on quantitative MRI parameters in the isodendritic core.

About PREVENT-AD

Full Acknowledgments

PREVENT-AD was launched in 2011 as a $13.5 million 7-year public-private partnership using funds provided by McGill University, the Fonds de Recherche du Québec-Santé (FRQS), an unrestricted research grant from Pfizer Canada, the Levesque Foundation, the Douglas Hospital Research Centre and Foundation, he Government of Canada, the Canada Fund for Innovation, the Canadian Institutes for Health Research, the Alzheimer Society of Canada and the Alzheimer Association. Private sector contributions are facilitated by the Development Office of the McGill University Faculty of Medicine and by the Douglas Hospital Research Centre Foundation (www.douglas.qc.ca).

The primary goal of PREVENT-AD is to test whether serial determination of multi-modal biomarkers of Alzheimer's disease may be measured and used in pre-symptomatic persons at high risk of subsequent AD dementia to trace the progression of the disease process and to measure effects of any potentially preventive treatment interventions. This work is intended to provide preliminary data regarding the probably efficacy and safety of potential new treatments for prevent of AD dementia.

The Founders of the program were John C. S. Breitner, MD, MPH, Judes Poirier, PhD, and Pierre Etienne, MD, Douglas Hospital Research Centre and Faculty of Medicine, McGill University, Montreal, QC, Canada. Program Current Director is Sylvia Villeneuve, PhD, the Co-Director is Judes Poirier, PhD, and the Study Coordinator is Jennifer Tremblay-Mercier, MSc. PREVENT-AD is the result of efforts of many other co-investigators from a range of academic institutions and private corporations, as well as an extraordinarily dedicated and talented clinical and technical assistant staff, students, and postdoctoral fellows. Subjects are recruited from the greater Montreal area and more distant locations in Quebec. For up-to-date information see https://prevent-alzheimer.net/?page_id=42&lang=en

Recruitment Strategy

To bring this population into our program, the main strategy was to send informative flyers about the PREVENT-AD program, in a bag called "publisac" containing different kind of publicity, weekly delivered to the Quebec population. People were invited to contact us by telephone or visit our web site (www.prevent-alzheimer.ca) if they were 55 or older and if they had a parent, brother or sister who has/had Alzheimer's disease. To date, 250,000 flyers were sent in 65 different areas Montreal, QC, CAN. The areas were chosen based on demographic statistics i.e. in areas with higher proportion of 55 years old or older, compared to the provincial average. People were also reached by various media coverage done on the Stop-AD Center and on principal investigators of the Center (television, radio, newspapers). At a smaller level, the program was presented in different academic institutions and nursing homes and flyers were distributed to medical professionals and general population in various occasions.

Full PREVENT-AD member list

The following people were part of the PREVENT-AD Research Group:

McGill University

Gabriel Aumont-Rodrigue, Mohamed Badawy, Sylvain Baillet, Julie Bailly, Andree-Ann Baril,Veronique Bohbot, Lianne Boisvert, John C.S. Breitner, Mallar Chakravarty, Louis Collins, Nolan-Patrick Cunningham, Mahsa Dadar, Samir Das, Marina Dauar-Tedeschi, Christine Dery, Simon Ducharme, Marie Josée Élie, Alan Evans, Alfonso Fajardo Valdez, Vladimir Fonov, Jonathan Gallago, Maiya R. Geddes, Rick Hoge, Louise Hudon, Yasser Ituria-Medina, Mohammadali Javanray, Gabriel Jean, Anne Labonte, Marc Lalancette, Lisa-Marie Munter, Laurence Maligne Bruneau, Julien Menes, Bery Mohammediyan, Gerhard Multhaup, Eugenia Nita Capota, Valentin Ourry, Cynthia Picard, Judes Poirier, Ting Qiu, Marc James Quesnel, Natasha Rajah, Jean-Michel Raoult, Jordana Remz, Pedro Rosa-Neto, Jean-Paul Soucy, R. Nathan Spreng, Frederic St-Onge, Elisabeth Sylvain, Christine Tardif, Jennifer Tremblay-Mercier, Stephanie Tullo, Etienne Vachon-Presseau, Sylvia Villeneuve, Yara Yakoub

University of Montreal

Pierre Bellec, Pierre Orban

Johns Hopkins University

Jeannie-Marie Leoutsakos

Supplementary Materials References

1. Theofilas, P., Dunlop, S., Heinsen, H. & Grinberg, L. T. Turning on the light within: subcortical nuclei of the isodentritic core and their role in Alzheimer's disease pathogenesis. *J Alzheimers Dis* **46**, 17–34 (2015).
2. Nobili, A. *et al.* Dopamine neuronal loss contributes to memory and reward dysfunction in a model of Alzheimer's disease. *Nat Commun* **8**, 14727 (2017).
3. Venneri, A. & De Marco, M. Reduced monoaminergic nuclei MRI signal detectable in pre-symptomatic older adults with future memory decline. *Sci Rep* **10**, 18707 (2020).
4. Burns, J. M., Galvin, J. E., Roe, C. M., Morris, J. C. & McKeel, D. W. The pathology of the substantia nigra in Alzheimer disease with extrapyramidal signs. *Neurology* **64**, 1397–1403 (2005).
5. De Marco, M. & Venneri, A. Volume and Connectivity of the Ventral Tegmental Area are Linked to Neurocognitive Signatures of Alzheimer's Disease in Humans. *J Alzheimers Dis* **63**, 167–180 (2018).
6. Levinson, S. *et al.* A structural connectivity atlas of limbic brainstem nuclei. *Front Neuroimaging* **1**, 1009399 (2022).
7. Bianciardi, M. *et al.* Toward an *In Vivo* Neuroimaging Template of Human Brainstem Nuclei of the Ascending Arousal, Autonomic, and Motor Systems. *Brain Connectivity* **5**, 597–607 (2015).
8. Edlow, B. L. *et al.* Neuroanatomic Connectivity of the Human Ascending Arousal System Critical to Consciousness and Its Disorders. *J Neuropathol Exp Neurol* **71**, 531–546 (2012).
9. Beliveau, V. *et al.* Functional connectivity of the dorsal and median raphe nuclei at rest. *NeuroImage* **116**, 187–195 (2015).

10. Ikuta, T. *et al.* Disconnectivity between Dorsal Raphe Nucleus and Posterior Cingulate Cortex in Later Life Depression. *Frontiers in Aging Neuroscience* **9**, (2017).
11. Kranz, G. S., Hahn, A., Savli, M. & Lanzenberger, R. Challenges in the differentiation of midbrain raphe nuclei in neuroimaging research. *Proceedings of the National Academy of Sciences* **109**, E2000–E2000 (2012).
12. Grinberg, L. T. *et al.* The dorsal raphe nucleus shows phospho-tau neurofibrillary changes before the transentorhinal region in Alzheimer's disease. A precocious onset? *Neuropathology and Applied Neurobiology* **35**, 406–416 (2009).
13. Amunts, K. *et al.* BigBrain: an ultrahigh-resolution 3D human brain model. *Science* **340**, 1472–1475 (2013).
14. Xiao, Y. *et al.* An accurate registration of the BigBrain dataset with the MNI PD25 and ICBM152 atlases. *Sci Data* **6**, 210 (2019).
15. Baker, K. G. *et al.* Cytoarchitecture of serotonin-synthesizing neurons in the pontine tegmentum of the human brain. *Synapse* **7**, 301–320 (1991).
16. Baker, K. G., Halliday, G. M. & Törk, I. Cytoarchitecture of the human dorsal raphe nucleus: HUMAN DORSAL RAPHE. *J. Comp. Neurol.* **301**, 147–161 (1990).
17. Ding, S.-L. *et al.* Comprehensive cellular-resolution atlas of the adult human brain. *Journal of Comparative Neurology* **524**, 3127–3481 (2016).
