# Supplementary material for: Neuromodulatory subcortical nucleus integrity is associated with white matter microstructure, tauopathy and APOE status
Source: Nat Commun. 2024 Jun 3;15:4706. doi: 10.1038/s41467-024-48490-z (PMC11148077; doi:10.1038/s41467-024-48490-z)
Supplement: Supplementary file 3 — Reporting Summary [file 41467_2024_48490_MOESM3_ESM.pdf]

nature portfolio

Corresponding author(s): Alfie Wearn, R. Nathan Spreng

Last updated by author(s): Apr 1, 2024

Reporting Summary

Nature Portfolio wishes to improve the reproducibility of the work that we publish. This form provides structure for consistency and transparency in reporting. For further information on Nature Portfolio policies, see our Editorial Policies and the Editorial Policy Checklist.

Statistics

For all statistical analyses, confirm that the following items are present in the figure legend, table legend, main text, or Methods section.

n/a	Confirmed	
☐	☒	The exact sample size (n) for each experimental group/condition, given as a discrete number and unit of measurement
☐	☒	A statement on whether measurements were taken from distinct samples or whether the same sample was measured repeatedly
☐	☒	The statistical test(s) used AND whether they are one- or two-sided<br>*Only common tests should be described solely by name; describe more complex techniques in the Methods section.*
☐	☒	A description of all covariates tested
☐	☒	A description of any assumptions or corrections, such as tests of normality and adjustment for multiple comparisons
☐	☒	A full description of the statistical parameters including central tendency (e.g. means) or other basic estimates (e.g. regression coefficient) AND variation (e.g. standard deviation) or associated estimates of uncertainty (e.g. confidence intervals)
☐	☒	For null hypothesis testing, the test statistic (e.g. F, t, r) with confidence intervals, effect sizes, degrees of freedom and P value noted<br>*Give P values as exact values whenever suitable.*
☒	☐	For Bayesian analysis, information on the choice of priors and Markov chain Monte Carlo settings
☒	☐	For hierarchical and complex designs, identification of the appropriate level for tests and full reporting of outcomes
☐	☒	Estimates of effect sizes (e.g. Cohen's d, Pearson's r), indicating how they were calculated

Our web collection on statistics for biologists contains articles on many of the points above.

Software and code

Policy information about availability of computer code

Data collection	No software was used for collection of data in this study.
Data analysis	This study utilised a number of open access resources. Multiparametric maps were processed using qMRLab (https://github.com/qMRLab/qMRLab) and hMRI toolbox (v 0.4.0) (https://hmri-group.github.io/hMRI-toolbox/) in MATLAB. Diffusion images were preprocessed using the MRtrix3 toolbox: https://www.mrtrix.org/. We ran PLS using an openly available MATLAB toolbox available to download here: https://github.com/McIntosh-Lab/PLS/. Partial correlations were performed using the openly available ppcor R package (v1.1) in R: https://CRAN.R-project.org/package=ppcor and visualised using the corrplot R package (v0.92). All other data presented in figures were organised and plotted using functions from the tidyverse R package (v2.0.0): https://github.com/tidyverse. The voxelwise salience maps were displayed using MRIcroGL: https://www.nitrc.org/projects/mricrogl. Source data are provided in this paper.

For manuscripts utilizing custom algorithms or software that are central to the research but not yet described in published literature, software must be made available to editors and reviewers. We strongly encourage code deposition in a community repository (e.g. GitHub). See the Nature Portfolio guidelines for submitting code & software for further information.

Data

Policy information about availability of data

All manuscripts must include a data availability statement. This statement should provide the following information, where applicable:

- Accession codes, unique identifiers, or web links for publicly available datasets
- A description of any restrictions on data availability
- For clinical datasets or third party data, please ensure that the statement adheres to our policy

This study used data from the PResymptomatic EValuation of Experimental or Novel Treatments for AD (PREVENT-AD) study. Data availability for PREVENT-AD is governed by the Open Access protocols. Please refer to https://douglas.research.mcgill.ca/prevent-alzheimer-program/ for more information about PREVENT-AD. Source data are provided in this paper.

Research involving human participants, their data, or biological material

Policy information about studies with human participants or human data. See also policy information about sex, gender (identity/presentation), and sexual orientation and race, ethnicity and racism.

Reporting on sex and gender	The research question did not include hypotheses based on sex or gender. Sex was included as a covariate in linear regression analyses but did not show any association with variables of interest. This is reported in the manuscript text.
Reporting on race, ethnicity, or other socially relevant groupings	We did not use any socially constructed or socially relevant variables in our analyses.
Population characteristics	We used a subset of 141 participants from the PREVENT-AD cohort for this study. After excluding 8 people for methodological reasons (e.g. registration failure), the total sample contained 133 individuals, with a mean [SD] age of 67.9 [5.3] years, including 95 females and a mean [SD] of 15.4 [3.5] years of education.
Recruitment	Data were collected as part of the PREVENT-AD longitudinal cohort in 2011-2017. Detailed recruitment and participant information is available in Tremblay-Mercier (2021)<br><br>Tremblay-Mercier, J., Madjar, C., Das, S., Pichet Binette, A., Dyke, S. O. M., Étienne, P., Lafaille-Magnan, M.-E., Remz, J., Bellec, P., Louis Collins, D., Natasha Rajah, M., Bohbot, V., Leoutsakos, J.-M., Iturria-Medina, Y., Kat, J., Hoge, R. D., Gauthier, S., Tardif, C. L., Mallar Chakravarty, M., … Breitner, J. C. S. (2021). Open science datasets from prevent-AD, a longitudinal cohort of pre-symptomatic alzheimer's disease. NeuroImage: Clinical, 31, 102733. https://doi.org/10.1016/j.nicl.2021.102733
Ethics oversight	Research ethics for the study was obtained from the Research Ethics Board of the Faculty of Medicine and Health Science at McGill University and/or the Comité d'éthique de la recherche du CIUSSS de l'ouest de l'ile de Montréal. Participants provided written informed consent.

Note that full information on the approval of the study protocol must also be provided in the manuscript.

Field-specific reporting

Please select the one below that is the best fit for your research. If you are not sure, read the appropriate sections before making your selection.

☒ Life sciences ☐ Behavioural & social sciences ☐ Ecological, evolutionary & environmental sciences

For a reference copy of the document with all sections, see nature.com/documents/nr-reporting-summary-flat.pdf

Life sciences study design

All studies must disclose on these points even when the disclosure is negative.

Sample size	We did not make use of statistical methods to determine sample size however our sample size is similar to, or greater than the samples used in the majority of MPM studies using a similar acquisition protocol (See Tabelow et al., 2019 for an overview).<br><br>Tabelow, K., Balteau, E., Ashburner, J., Callaghan, M. F., Draganski, B., Helms, G., Kherif, F., Leutritz, T., Lutti, A., Phillips, C., Reimer, E., Ruthotto, L., Seif, M., Weiskopf, N., Ziegler, G., & Mohammadi, S. (2019). HMRI – a toolbox for quantitative MRI in Neuroscience and Clinical Research. NeuroImage, 194, 191–210. https://doi.org/10.1016/j.neuroimage.2019.01.029
Data exclusions	Participants were selected from the larger PREVENT-AD cohort if they had Multiparametric mapping imaging data as well as diffusion data. 8 subjects were excluded due to poor image alignment between modalities, or to standard template space, and were therefore not able to have accurate data extracted from the regions of interest. One additional subject did not have genotype data available so was excluded from APOE4 analysis.
Replication	We cannot conduct a direct replication at this time as there is not another dataset that contains the necessary MRI sequences (MPM, mutlishell diffusion) and CSF biomarkers in a cohort of older adults. The PAD cohort is longitudinal and we hope to replicate these findings at a

future time point.

Randomization	This study did not contain different experimental conditions.
Blinding	This study did not contain different experimental conditions so blinding was not possible. The staff involved in data collection were not involved in data analysis.

Reporting for specific materials, systems and methods

We require information from authors about some types of materials, experimental systems and methods used in many studies. Here, indicate whether each material, system or method listed is relevant to your study. If you are not sure if a list item applies to your research, read the appropriate section before selecting a response.

Materials & experimental systems

n/a	Involved in the study
☒	☐ Antibodies
☒	☐ Eukaryotic cell lines
☒	☐ Palaeontology and archaeology
☒	☐ Animals and other organisms
☒	☐ Clinical data
☒	☐ Dual use research of concern
☒	☐ Plants

Methods

n/a	Involved in the study
☒	☐ ChIP-seq
☒	☐ Flow cytometry
☐	☒ MRI-based neuroimaging

Plants

Seed stocks	*Report on the source of all seed stocks or other plant material used. If applicable, state the seed stock centre and catalogue number. If plant specimens were collected from the field, describe the collection location, date and sampling procedures.*
Novel plant genotypes	*Describe the methods by which all novel plant genotypes were produced. This includes those generated by transgenic approaches, gene editing, chemical/radiation-based mutagenesis and hybridization. For transgenic lines, describe the transformation method, the number of independent lines analyzed and the generation upon which experiments were performed. For gene-edited lines, describe the editor used, the endogenous sequence targeted for editing, the targeting guide RNA sequence (if applicable) and how the editor was applied.*
Authentication	*Describe any authentication procedures for each seed stock used or novel genotype generated. Describe any experiments used to assess the effect of a mutation and, where applicable, how potential secondary effects (e.g. second site T-DNA insertions, mosiacism, off-target gene editing) were examined.*

Magnetic resonance imaging

Experimental design

Design type	Correlational study design
Design specifications	*Specify the number of blocks, trials or experimental units per session and/or subject, and specify the length of each trial or block (if trials are blocked) and interval between trials.*
Behavioral performance measures	No measures of behaviour were examined in this study

Acquisition

Imaging type(s)	Structural, Diffusion, Quantitative
Field strength	3T
Sequence & imaging parameters	For MPM, three multi-echo gradient echo sequences were acquired (1mm isotropic resolution, TA=17:30) with predominant weighting for: T1 (TR=18ms, 6 echoes, TE=2.16-14.81ms, FA 20), MT (TR=27ms, 6 echoes, TE=2.04-14.89ms, echo-spacing=2.57ms, FA 6) or PD (TR=27ms, 8 echoes, TE=2.04-22.20ms, echo-spacing=2.57ms, FA 6).<br>B1+ transmit field maps were acquired using two spin-echo echo-planar sequences with different flip angles (60, 120) and otherwise identical parameters (2x2x4mm resolution, TR/TE=4010/46 ms, TA=1:08).<br>Maps for MTsat, R1, R2* and PD were corrected for B1- receive field inhomogeneities, calculated from two PD-weighted turbo-flash sequences acquired using either the body coil or 32-channel head coil, with otherwise identical acquisition parameters (2mm isotropic resolution, TR/TE=344/1.55 ms, FA=3, TA = 0:35).<br><br>Diffusion-weighted imaging was performed with a 2mm isotropic spin-echo echo-planar imaging sequence with 109 measurements (isotropically spaced around a sphere) across three shells with the following b-values: 7 b=300, 29

b=1000 and 64 b=2000 s/mm2, as well as 9 b=0 images (TR/TE=3000/66ms, posterior-anterior phase encoding, TA=5:49). Five additional b=0 images were acquired with reversed phase encoding direction and otherwise identical sequence parameters for subsequent distortion correction.

Area of acquisition

All scans used covered whole brain

Diffusion MRI ☒ Used ☐ Not used

Parameters

Diffusion-weighted imaging was performed with a 2mm isotropic spin-echo echo-planar imaging sequence with 109 measurements (isotropically spaced around a sphere) across three shells with the following b-values: 7 b=300, 29 b=1000 and 64 b=2000 s/mm2, as well as 9 b=0 images (TR/TE=3000/66ms, posterior-anterior phase encoding, TA=5:49). Five additional b=0 images were acquired with reversed phase encoding direction and otherwise identical sequence parameters for subsequent distortion correction.

Preprocessing

Preprocessing software

B1+ maps were created in qMRLab using the double-angle method with a sixth-order polynomial smoothing kernel. All further processing of MPM maps was performed using the hMRI toolbox (v 0.4.0). Parameter maps were denoised using ANTS DenoiseImage.

Diffusion images were preprocessed using the MRtrix3 toolbox. Images were denoised (dwidenoise), corrected for eddy, susceptibility and motion artefacts (dwifslpreproc) and upsampled to 1mm isotropic resolution (mrgrid). Upsampled images were brain-extracted (bet2), corrected for bias-field artefact using the ANTs algorithm (N4) of the dwibiascorrect MRtrix function. Bias field corrected diffusion-weighted data were fitted to the NODDI model using the python implementation of Accelerated Microstructure Imaging via Convex Optimization (AMICO).

Normalization

Rregistrations of MPM maps were performed using ANTS (antsRegistrationSyNQuick.sh). For each subject, the denoised R1 map was registered (affine) to the denoised MPRAGE. Then, each subject's MPRAGE was normalized to the standard-space T1w template using a previously described two-step process. First, rigid then affine registrations were calculated with the denoised MPRAGE as the moving image and the MNI template as the fixed image. This created an approximate alignment between the images. Second, this approximately aligned MPRAGE was registered to the template image again, this time using subsequent rigid, affine then non-linear (SyN) warps, the latter stage of which used a manually defined registration mask of the brainstem, midbrain and basal forebrain area (Supplementary Fig. 2). This ensured good quality alignment within this region (confirmed by visual inspection of all cases). As a final step, warps were inverted and concatenated to transform MNI-space ROIs into each subject's native MPM-space (with nearest neighbor interpolation).

Normalization of NODDI map to MNI space: Upsampled NODDI maps and probabilistic white-matter masks were co-registered with MPM maps using FSL 'flirt' (rigid body registration, using b=0 volumes as the moving image and PD maps as a reference image due to similar contrast). MTsat maps were segmented into gray matter and white matter tissue compartments, then deformation fields were estimated between native space and MNI (2009c) space using the 'Shoot' toolbox in SPM12. The resulting deformation fields were then applied to warp the MPM-space NODDI maps and white-matter masks, thereby bringing them into MNI template space.

Normalization template

For MPM, and isodendritic core nuclei ROI data extraction: MNI space (ICBM 2009b 0.5mm T1-weighted)

For normalizing whole-brain NODDI maps - MNI ICBM 2009c Asym 1mm T1-weighted (default of 'shoot' toolbox)

Noise and artifact removal

ANTS DenoiseImage was used to denoise MPRAGE and MTsat images prior to calculating warps and MTsat values. Images were also visualled inspected for artefacts that would significantly influence signal derived from ROIs.

Volume censoring

n/a

Statistical modeling & inference

Model type and settings

To explore the relationship of IdC with demographic variables and CSF biomarkers we ran partial correlations, correcting for demographic covariates (age, sex, years of education).

We use partial least squares (PLS) analysis to assess the multivariate spatial patterns of covariance between four MPM-derived measures of microstructural integrity (R1, MTsat, R2*, PD) in the four IdC nuclei (LC, DR, VTA, NbM) and three NODDI measures of white matter microstructure (NDI, ODI, FW). Each NODDI-parameter map had age and MPM-parameters of the pontine ROI regressed-out prior to analysis and was entered as a separate condition in a single PLS analysis. We did not examine MPM-derived measures across the whole-brain white matter in order to focus our findings in the context of the NODDI model. The PLS was run with 1000 permutations to determine the significance of each LV, and 1000 bootstraps to determine overall reliability of each voxel's contribution to each LV by calculating the standard error of each voxel's salience value. Only significant ($p<0.05$) LVs and voxels with bootstrap ratios >|2| (calculated as the ratio of each voxel's salience to its standard error) were interpreted. Bootstrap ratios are equivalent to a z statistic and values > |2| are roughly equivalent to a p-value < 0.05. Clusters smaller than 32 voxels (default of MRIcroGL viewing software) were also excluded. Individual effects of IdC MPM parameters were deemed significant if 95% bootstrap confidence intervals on correlation coefficients did not overlap with zero. In order to visualize which NODDI parameter contributed most to each LV, we summed design scores across all IdC-MPM parameters for each NODDI condition.

We used regression models to test whether PLS brain scores were related to Alzheimer's pathology. For each model the brain score served as the dependent variable and CSF biomarker concentration (either pTau181 or Aß42) served as the independent predictor variables, with sex and years of education as covariates (age was already residualized out of the NODDI maps, so was not included here as well).

Effect(s) tested

Multivariate latent patterns in the correlation matrix between voxelwise white matter microstructure (3x NODDI parameters) and MPM-derived microstructure measures in all four nuclei of the isodendritic core.

Specify type of analysis: ☐ Whole brain ☐ ROI-based ☒ Both

Anatomical location(s)

MPM measures were derived from 4 nuclei of the isodendritic core (and one control region). Previously validated probablistic atlases were used to define LC (thresholded at 10%), VTA (thresholded at 25%) and NbM (thresholded at 60%) (Supplementary Fig. 1). DR was manually defined by placing a sphere of 3mm radius at MNI coordinates: x:0 y:-27 z:-9. The borders were edited slightly to restrict overlap into cerebral aqueduct (total volume 32mm3). This position was chosen by cross-validating previous histological sources with novel histological information provided by BigBrain histological atlas spatially localized to MNI-space. We also defined a bilateral spherical pontine ROI that did not overlap with any part of the IdC as a methodological control region (Supplementary Fig. 3).

NODDI measures were examined in every voxel of white matter across the brain. A probabilistic mask of each individual's normal-appearing white matter was made using the 5ttgen function of MRtrix3. White matter masks warped to MNI space were averaged across individuals and thresholded at 0.95.

Statistic type for inference

(See Eklund et al. 2016)

associations between a priori defined ROIs and voxelwise white matter were examined in a robust, multivariate analysis (PLS).

Correction

A benefit of Partial Least Squares analysis is that it does not necessitate correction for multiple comparisons across variables used as inputs. 'Significant' areas of effect are defined by bootstrapping and permutation analysis to calcualte bootstrap ratios and confidence intervals for all effects.

Models & analysis

n/a	Involved in the study
☒	☐ Functional and/or effective connectivity
☒	☐ Graph analysis
☐	☒ Multivariate modeling or predictive analysis

Multivariate modeling and predictive analysis

We use partial least squares (PLS) analysis to assess the multivariate spatial patterns of covariance between four MPM-derived measures of microstructural integrity (R1, MTsat, R2*, PD) in the four IdC nuclei (LC, DR, VTA, NbM) and three NODDI measures of white matter microstructure (NDI, ODI, FW). This reduces the dimensionality of the correlation matrix to latent variables that describe the most covariance.

The PLS was run with 1000 permutations to determine the significance of each LV, and 1000 bootstraps to determine overall reliability of each voxel's contribution to each LV by calculating the standard error of each voxel's salience value. Only significant (p<0.05) LVs and voxels with bootstrap ratios >|2| (calculated as the ratio of each voxel's salience to its standard error) were interpreted. Bootstrap ratios are equivalent to a z statistic and values > |2| are roughly equivalent to a p-value < 0.05.
